# The effect of age at first dose of a measles-containing vaccine on measles humoral immunity: a retrospective cohort study, Canada, 2014 to 2016

**DOI:** 10.2807/1560-7917.ES.2026.31.45.2600105

**Published:** 2026-11-12

**Authors:** Archchun Ariyarajah, Natasha S Crowcroft, Kevin A Brown, John Wang, Jeffrey C Kwong, Shelly Bolotin

**Affiliations:** 1 Dalla Lana School of Public Health, University of Toronto, Toronto, Canada; 2 Centre for Vaccine Preventable Diseases, University of Toronto, Toronto, Canada; 3 ICES, Toronto, Canada; 4 Public Health Agency of Canada, Ottawa, Canada; 5 Public Health Ontario, Toronto, Canada; 6 Department of Family and Community Medicine, University of Toronto, Toronto, Canada; 7 Department of Laboratory Medicine and Pathobiology, University of Toronto, Toronto, Canada; * These authors contributed equally to this work and share last authorship.

**Keywords:** measles, age at vaccination, serology

## Abstract

**BACKGROUND:**

Canada eliminated measles in 1998; however, large outbreaks have been recently reported, and elimination status was lost in 2025.

**AIM:**

We aimed to estimate the effect of age at first dose of measles-containing vaccine (MCV1) on measles IgG concentration and seronegativity among two-dose recipients nearly two decades after vaccination.

**METHODS:**

We conducted a retrospective cohort study of 10,766 twice-vaccinated individuals tested for measles IgG from 2014 to 2016 in Ontario, Canada. We predicted measles IgG concentration and seronegativity using thresholds of < 275 mIU/mL, < 200 mIU/mL and ≤ 120 mIU/mL.

**RESULTS:**

A 1-month increase in age at MCV1 (between 12 and 15 months) was associated with 13.7% (95% confidence interval (CI): 7.1–20.8) higher predicted mean measles IgG concentration for females born after elimination and 9.2% (95% CI: 6.8–11.6) for females born before elimination. Depending on the seronegativity threshold and the measles elimination status at birth, a 1-month increase in age at MCV1 (between 12 and 15 months) was associated with lower odds ratio of seronegativity ranging from 0.74 (95% CI: 0.63–0.85) to 0.87 (95% CI: 0.57–1.27). Using the lowest threshold (≤ 120 mIU/mL) and the longest follow-up period (24 years post-MCV1), we predicted that 4.1% (95% CI: 2.7–6.1) of females vaccinated with MCV1 at 15 months of age would be seronegative, compared with 9.6% (95% CI: 7.4–12.3) of those vaccinated at 12 months.

**DISCUSSION:**

Delaying MCV1 to older ages (13–15 months) may result in an absolute decrease as large as 6% in the percentage of seronegative individuals, potentially increasing the likelihood of maintaining herd immunity.

**Key public health message**

**What did you want to address in this study and why?**

Younger age at first measles vaccine dose (12 months of age) protects infants sooner, but administering it later (13–15 months of age) may provide longer-lasting immunity. There is little evidence on the long-term immunity by age at first dose in countries with no sustained measles transmission. Using blood test results from Canada, we evaluated whether antibody levels are higher when vaccinated at 13–15 vs 12 months in people who have been vaccinated twice.

**What have we learnt from this study?**

Nearly two decades after their first measles vaccine dose, adults who had received two doses in childhood had 1.1 to 1.5 times higher measles antibody concentrations if they had their first measles dose at older ages (13–15 months) instead of the recommended age of 12 months. They were also less likely to have antibody concentrations below the threshold of protection.

**What are the implications of your findings for public health?**

These findings highlight a trade-off for the timing of measles vaccination: earlier vaccination may reduce short-term risk of measles in infants, while later vaccination may improve long-term protection. With measles resurging and countries such as Canada recently losing their measles elimination status, countries may need to weigh the benefits and risks of the current recommended age at first dose (12 months) compared with delaying it by a few months.

## Background

Two-dose measles vaccination programmes have reduced measles cases globally [1]. Some countries have achieved measles elimination, which is the absence of endemic transmission for ≥ 12 months [1]. However, suboptimal vaccine coverage, driven by lower vaccine acceptance and poor accessibility during the COVID-19 pandemic, have led to a measles resurgence, and several countries have re-established endemic transmission after achieving measles elimination [1-3].

Measles-containing vaccine (MCV) is highly effective, with 95% of vaccinated individuals seroconverting after the first dose (MCV1) given at 12 months or older [4]. The second dose (MCV2) ensures seroconversion for non-responders, protecting 99% of vaccinated individuals [4]. The optimal age for MCV1 balances the risks of vaccinating too early, which can lead to vaccine failure due to maternal antibodies and an immature immune system, and vaccinating too late, which leaves young children vulnerable to infection and complications [4,5]. While most measles-eliminated countries schedule MCV1 at 12 months, some schedule it at 15–18 months to improve long-term immunity [6,7]; studies show that vaccination with MCV1 at older ages often, but not always, results in higher measles IgG concentration [8,9]. Waning immunity in individuals vaccinated at younger ages has been observed in measles-eliminated settings, raising concerns about their risk of measles if exposed [6,10]. Canada eliminated measles in 1998 but lost its elimination status in November 2025 [3,11]. In Ontario, MCV1 was consistently scheduled at 12 months of age from 1993 to 2016. The MCV2 dose was introduced in 1996 and was scheduled at 4–6 years of age from 1996 to 2004 and at 18 months from 2005 to July 2011 before being moved to ages 4–6 years again in August 2011 [12].

This study aimed to estimate the effect of age at MCV1 on measles IgG concentration and seronegativity among two-dose recipients in Ontario to determine if vaccination at older ages (13–15 months) results in more robust long-term humoral immunity.

## Methods

### Study design and data sources

We used a retrospective cohort study design to measure the effect of age at MCV1 on measles IgG concentration and seronegativity among two-dose recipients. We used serology and RT-PCR test results from Public Health Ontario (PHO) laboratories, which conduct occupational, prenatal and outbreak-related measles testing. We linked diagnostic test results for measles IgG, IgM and RT-PCR conducted in the period of 2014 to 2016 to the Ontario Registered Persons Database housed at ICES (formerly the Institute for Clinical Evaluative Sciences). As an independent, non-profit research institute with legal status under Ontario’s health information privacy law, ICES may collect and analyse healthcare and demographic data, without consent, for health system evaluation and improvement. These datasets were linked using unique encoded identifiers and analysed at ICES. A description of the included databases for sociodemographic and health-related information is appended in Supplementary Table S1. We identified individuals with immunocompromising conditions using a pre-established algorithm [13]. Due to data availability, only migrants whose place of first arrival in Canada was Ontario could be identified.

### Outcome

Measles IgG antibodies are markers of protective immunity. Public Health Ontario measured measles IgG concentration in serum samples using enzyme linked immunosorbent assay (ELISA) test kits manufactured by Euroimmun AG (Lübeck, Germany), who report 100% test sensitivity and specificity [14]. We rounded measles IgG concentrations to the nearest integer values to use count models. Since the test only reports values within the range of 50–5,000 mIU/mL, we converted values reported as < 50 mIU/mL or > 5,000 mIU/mL to 50 mIU/mL and 5,000 mIU/mL, respectively. The evidence for the often cited > 120 mIU/mL correlate of protection for measles is limited [15,16]; therefore, we defined measles seronegativity according to three thresholds: (i) < 275 mIU/mL, according to the manufacturer’s recommendations [14]; (ii) < 200 mIU/mL, where indeterminate test results (200 to < 275 mIU/mL) are considered seropositive; and (iii) ≤ 120 mIU/mL. Detailed data for the distribution of measles IgG concentration are appended in Supplementary Figure S1.

### Exposure

Age at MCV1 in months was calculated using physician immunisation billing codes, rounded to one decimal place, and ranged from 12.0 to 14.9 months. Physicians bill the Ontario Health Insurance Plan (OHIP) using these codes, which include general codes that do not differentiate between any vaccines (introduced in January 1990) and a measles-mumps-rubella (MMR) vaccine-specific code (introduced in September 2011) [17]. All Ontario residents are eligible for OHIP except for recently arrived (< 3 months) migrants. To classify MCV1, we prioritised the first MMR vaccine-specific billing code closest to 12 months of age for individuals vaccinated from September 2011 to December 2016. Detailed information on the algorithm to identify MCV1 is appended in Supplementary Table S2. If individuals did not have MCV1 classified, we then used general immunisation billing codes to identify MCV1 among individuals vaccinated from January 1993 to December 2016. Since general immunisation billing codes cannot identify specific vaccines, a single code could represent MCV1 or another vaccine scheduled at the same age. To minimise misclassification, we created an algorithm to classify MCV1 only when the number of general immunisation billing codes was at least equal to the total number of vaccines concurrently recommended according to Ontario’s vaccination schedules from 1993 to 2016. For example, when MCV1 and two other vaccines were scheduled at 12 months, we only classified MCV1 if we observed at least three general billing codes within a 3-day period (the smallest observable time frame without compromising privacy). We did not classify MCV1 if additional general immunisation codes appeared at different time points between ages 12 to 15 months since we could not determine which code represented MCV1.

### Covariates

We controlled for time since MCV1 (continuous) and for sex (binary), both identified a priori as potential confounders based on evidence of their influence on measles immunity and delayed vaccination [10,18,19].

### MCV2 recipients

Individuals were classified as MCV2 recipients when the minimum number of general billing codes were recorded, consistent with the recommended schedule for MCV2 and co-administered vaccines. Detailed information on the algorithm to identify MCV2 is appended in Supplementary Table S3. However, since general billing codes have not been validated to classify MCV2 vaccination, we sought to identify MCV2 recipients and not ascertain the precise age at MCV2 vaccination.

### Study population

We excluded test results with incomplete or inconsistent records (e.g. missing birth date, sex or postal code, or death before the test date) and individuals not residing in Ontario. From the remaining test results, we excluded samples from individuals with evidence of previous or current measles infection by testing positive or indeterminate for measles IgM or RT-PCR, only if these data were available. If no data were available for measles IgM or RT-PCR, individuals classified as not having had a measles infection. We excluded unresolvable duplicate records, and samples that had been tested twice and had different quantitative test results. Among individuals who were sampled for measles IgG multiples times over the study period, we only included the earliest test result and excluded subsequent test results to minimise selection bias, given that re-testers are more likely to be healthcare workers. We excluded samples from individuals too young to be eligible for routine MCV1, individuals without MCV1 classified through immunisation billing codes, and individuals with serological testing dates preceding the immunisation billing date. We excluded identified migrants, since an earlier vaccination according to another country's schedule would not be captured in our databases. A small minority of our sample may include migrants who initially arrived in another province or territory and later moved to Ontario before receiving MCV1. To ensure our sample included individuals eligible for routine MCV2 under Ontario’s two-dose vaccination programme, we excluded those born before 1992, since MCV2 was introduced in 1996 for 4-year-olds in Ontario. Given the high vaccination coverage (≥ 94% among 17-year-olds from 2013 to 2016) and that immunisation for both doses is required for school attendance (with some exemptions allowed), most MCV2-eligible individuals probably received MCV2 [20]. We excluded individuals living with immunocompromising conditions, since their vaccine-derived immunity may wane more quickly compared with immunocompetent individuals [21]. The detailed number of excluded test results are appended in Supplementary Figure S2.

### Statistical analyses

We fit a multivariable negative binomial model for measles IgG concentration and a logistic model for the percentage seronegative for measles IgG, controlling for sex and time since MCV1. We ran separate analyses for cohorts born before and after elimination (in 1998) since the likelihood of measles exposure after vaccination is higher in the former cohort. Based on the regression model, we predicted the mean measles IgG concentration and the percentage seronegative by age at MCV1 for females, the most prevalent sex in our sample, and at 18 years and 24 years post-MCV1, the longest follow-up time for cohorts born after and before elimination, respectively. In addition, we predicted the percentage seronegative for age-specific populations (2, 7, 12 and 17 years) with sex parity and born after elimination. We estimated age by adding 1 year to the time since MCV1.

### Sensitivity analyses

Sensitivity analysis 1 (SA1) used an algorithm with lower specificity and higher sensitivity compared with the main analysis, since physicians may not have billed separate codes for each vaccine. We classified the first general billing code at the youngest age within 12.0–14.9 months of age as MCV1, irrespective of any additional billing codes at a later age. To evaluate the increased sensitivity, we compared the proportion of serum samples with classified MCV1 across different periods.

Sensitivity Analysis 2 (SA2) included individuals who were not classified as MCV2 recipients based on physician billing codes to improve the precision of our estimates, based on the assumption that most would have received the MCV2 given the high vaccination coverage. This approach allowed us to retain an additional 22,791 individuals who had been excluded in the main analysis. Detailed information for SA1 and SA2 is appended in Supplementary Figures S3 and S4, and Tables S3 and S4.

Sensitivity Analysis 3 (SA3) included estimates from the main analysis but predicted for males.

## Results

### Sample description

Our study sample included 10,766 individuals, where 8,041 (74.7%) were female and 10,721 (99.6%) were aged 12–25 years (Table 1). The predominance of females in our sample may reflect occupational health testing requirements, particularly among healthcare workers who are majority female, and is supported by sex parity observed among younger individuals [22]. Regarding MCV1 timing, 7,113 (66.1%) individuals were vaccinated at age 12–12.9 months, followed by 2,403 (22.3%) at age 13–13.9 months, and 1,250 (11.6%) at age 14–14.9 months. Delayed vaccination did not vary by neighbourhood-level income quintile. In our sample, 6,478 (60.2%) individuals had a documented reason for testing. Among individuals with a known reason for testing (physicians could select more than one), 5,423 (83.7%) were tested for immune status, 1,348 (20.8%) for diagnostic purposes, and 72 (1.1%) for prenatal testing. Testing was probably not related to an outbreak since Ontario reported an annual average of 16 cases in the years 2014 to 2016 [23]. Detailed data for age and sex distribution in the sample, and age at MCV1 by neighbourhood-level quintile are appended in Supplementary Figures S5 and S6.

**Table 1:** Characteristics of twice-vaccinated participants, Ontario, Canada, 2014–2016 (n = 10,766)

Category	All participants		Born after elimination^a^		Born before elimination^a^	
	n	%	n	%	n	%
Total	10,766	100.0	1,426	100.0	9,340	100.0
Sex						
Female	8,041	74.7	1,001	70.2	7,040	75.4
Male	2,725	25.3	425	29.8	2,300	24.6
Age at sample collection (years)						
1–5	8	0.1	8	0.6	0	0.0
6–11	37	0.3	37	2.6	0	0.0
12–19	5,567	51.7	1,381	96.8	4,186	44.8
20–25	5,154	47.9	0	0.0	5,154	55.2
Median (interquartile range)	19.8 (18.3–21.7)		17.0 (16.1–18.0)		20.3 (18.9–21.9)	
Birth cohort (by elimination status^a^ and vaccination schedule)						
1992–1997	9,340	86.8	0	0.0	9,340	100.0
1998–2004	1,391	12.9	1,391	97.5	0	0.0
2005–2011	35	0.3	35	2.5	0	0.0
2012–2016	0	0.0	0	0.0	0	0.0
Age at MCV1 (months)						
12	7,113	66.1	999	70.1	6,114	65.5
13	2,403	22.3	300	21.0	2,103	22.5
14	1,250	11.6	127	8.9	1,123	12.0
Median (interquartile range)	12.6 (12.2–13.2)		12.5 (12.2–13.1)		12.6 (12.2–13.2)	
Neighbourhood income quintile						
1 (lowest)	1,818	16.9	224	15.7	1,594	17.1
2	2,105	19.6	244	17.1	1,861	19.9
3	2,090	19.4	313	21.9	1,777	19.0
4	2,237	20.8	320	22.4	1,917	20.5
5 (highest)	2,452	22.8	318	22.3	2,134	22.9
Missing values	64	0.6	7	0.5	57	0.6

MCV1: first dose of measles-containing vaccine.

^a^ Canada eliminated measles in 1998.

### Predicted mean measles IgG concentration

Among two-dose recipients born after elimination, a 1-month increase in age at MCV1 (between 12 and 15 months) was associated with a 13.7% (95% confidence interval (CI): 7.1–20.8) increase in mean measles IgG concentration, controlling for sex and time since MCV1 (Table 2). When predicting for females born after elimination and serologically tested at 18 years post-MCV1, higher predicted mean measles IgG concentrations were observed for those vaccinated with MCV1 at 13.0 months (714 mIU/mL; 95% CI: 665–767 mIU/mL), 14.0 months (812 mIU/mL; 95% CI: 732–900 mIU/mL), and 15.0 months (923 mIU/mL; 95% CI: 793–1,075 mIU/mL) compared with 12.0 months (628 mIU/mL; 95% CI: 579–682 mIU/mL) (Figure 1). Detailed data for predicted mean measles IgG concentrations and percentage seronegative are appended in Supplementary Table S6.

**Table 2:** Estimated association per 1-month increase in age at MCV1 (between 12 and 15 months) on predicted mean measles IgG concentration and odds of measles seronegativity among twice-vaccinated individuals, Ontario, Canada, 2014–2016 (n = 10,766)

Outcome	Measles elimination status at birth^a^	
	Born after elimination	Born before elimination
Per cent change (95% CI)		
Predicted mean measles IgG concentration	13.7 (7.1–20.8)	9.2 (6.8–11.6)
OR (95% CI)		
Measles seronegativity (< 275 mIU/mL)	0.87 (0.73–1.03)	0.82 (0.77–0.88)
Measles seronegativity (< 200 mIU/mL)	0.84 (0.66–1.05)	0.78 (0.72–0.85)
Measles seronegativity (≤ 120 mIU/mL)	0.87 (0.57–1.27)	0.74 (0.63–0.85)

CI: confidence interval; IgG: immunoglobulin G; OR: odds ratio.

^a^ Canada eliminated measles in 1998.

**Figure 1:**
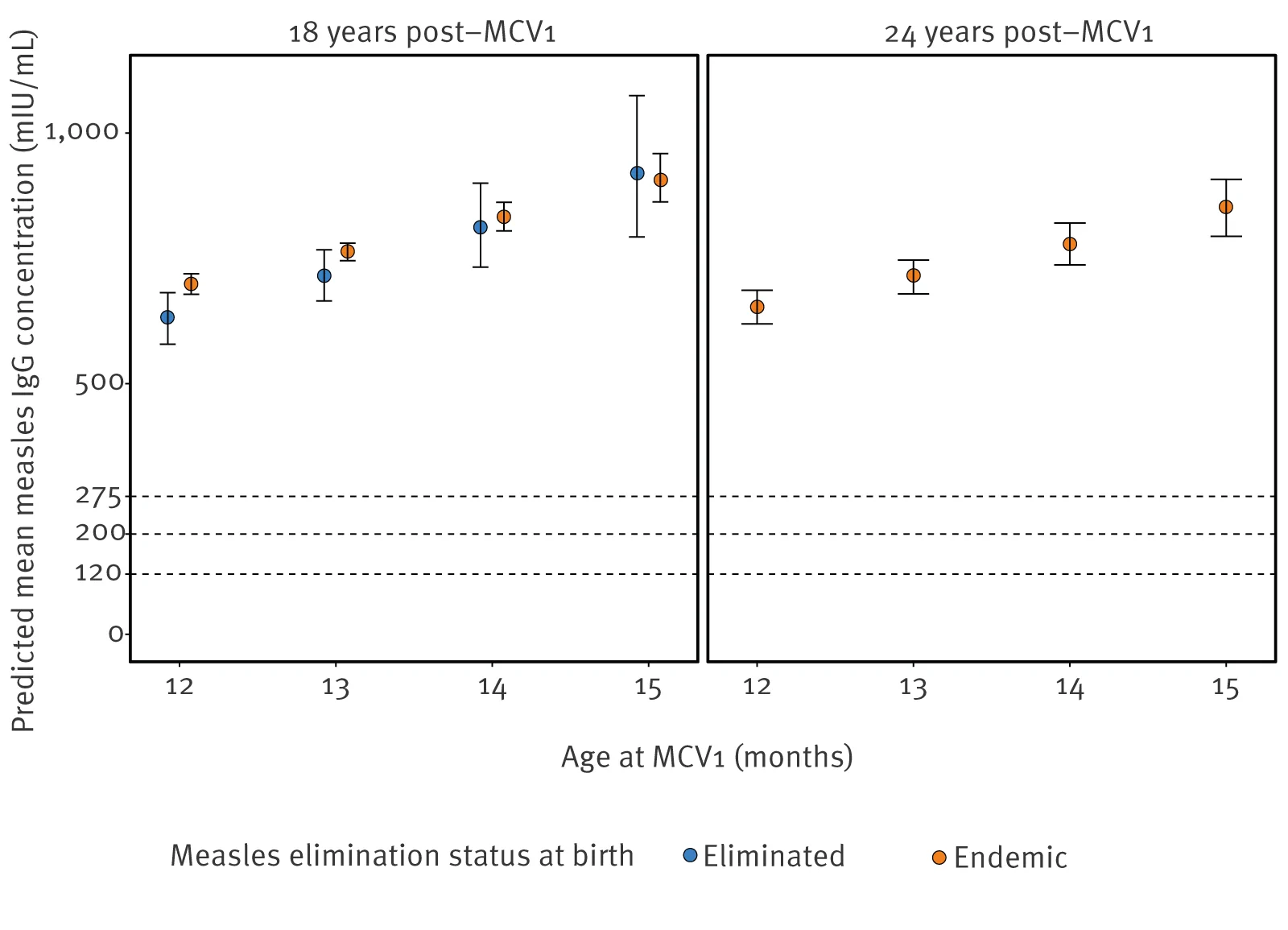
Predicted measles IgG concentration for twice-vaccinated females 18 and 24 years after first dose of measles-containing vaccine by age at first dose and measles elimination status at birth, Ontario, Canada, 2014–2016 (n = 10,766)

IgG: immunoglobulin G; MCV1: first dose of measles-containing vaccine.

Among two-dose recipients born before elimination, a 1-month increase in age at MCV1 (between 12 and 15 months) was associated with a 9.2% (95% CI: 6.8–11.6) increase in mean measles IgG concentration, controlling for sex and time since MCV1 (Table 2). When predicting for females born before elimination and serologically tested at 18 years post-MCV1, a similar pattern was observed where females vaccinated with MCV1 at 13.0–15.0 months had higher predicted measles IgG concentrations than those vaccinated at 12.0 months, with 763 mIU/mL (95% CI: 745–780 mIU/mL) at 13.0 months, 833 mIU/mL (95% CI: 805–862 mIU/mL) at 14.0 months, 909 mIU/mL (95% CI: 862–959 mIU/mL) at 15.0 months, compared with 698 mIU/mL (95% CI: 678–719 mIU/mL) at 12.0 months (Figure 1).

Among females born before elimination, the trend of higher predicted mean IgG concentrations among those vaccinated with MCV1 at 13.0–15.0 months persisted at 24 years post-MCV1, the longest follow-up period. Predicted mean concentrations were lowest at 652 mIU/mL (95% CI: 619–686 mIU/mL) for those vaccinated at 12.0 months and highest at 849 mIU/mL (95% CI: 794–908 mIU/mL) for those vaccinated at 15.0 months (Figure 1). The predicted mean concentrations at 24 years post-MCV1 were ca 7% lower than those observed at 18 years post-MCV1 for females born before elimination, comparing across equivalent ages at MCV1. The underlying data for observed geometric mean measles IgG concentrations by age at MCV1 and time since MCV1 are additionally provided in Supplementary Table S7.

### Measles seronegativity

A 1-month increase in age at MCV1 (between 12 and 15 months) was associated with a lower odds ratio (OR) for measles seronegativity using all three thresholds in our analysis of two-dose recipients, controlling for sex and time since MCV1. Among those born after elimination, a 1-month increase in age at MCV1 (between 12 and 15 months) was associated with an OR of 0.87 (95% CI: 0.73–1.03) using the < 275 mIU/mL threshold, 0.84 (95% CI: 0.66–1.05) using the < 200 mIU/mL threshold, and 0.87 (95% CI: 0.57–1.27) using the ≤ 120 mIU/mL threshold; however, these decreases were not statistically significant. Among those born before elimination, a 1-month increase in age at MCV1 (between 12 and 15 months) was associated with an OR of 0.82 (95% CI: 0.77–0.88) using the < 275 mIU/mL threshold, 0.78 (95% CI: 0.72–0.85) using the < 200 mIU/mL threshold, and 0.74 (95% CI: 0.63–0.85) using the ≤ 120 mIU/mL threshold; these decreases were statistically significant.

Among females born after elimination, the predicted percentage seronegative at 18 years post-MCV1 was lower among those vaccinated with MCV1 at age 13–15 months compared with females vaccinated at age 12 months, regardless of the threshold for seronegativity (Figure 2). Using the threshold of < 275 mIU/mL, the predicted percentage seronegative ranged from 30.5% (95% CI: 25.8–35.8) for those vaccinated at age 12 months to 22.3% (95% CI: 15.4–31.1) for those vaccinated at age 15 months. Using the < 200 mIU/mL threshold, it ranged from 18.7% (95% CI: 14.5–23.9) for vaccination at age 12 months to 12.0% (95% CI: 6.9–20.0) for vaccination at age 15 months. Using the ≤ 120 mIU/mL threshold, it ranged from 4.9% (95% CI: 3.0–8.0) for vaccination at age 12 months to 3.3% (95% CI: 1.2–8.7) for vaccination at age 15 months.

**Figure 2:**
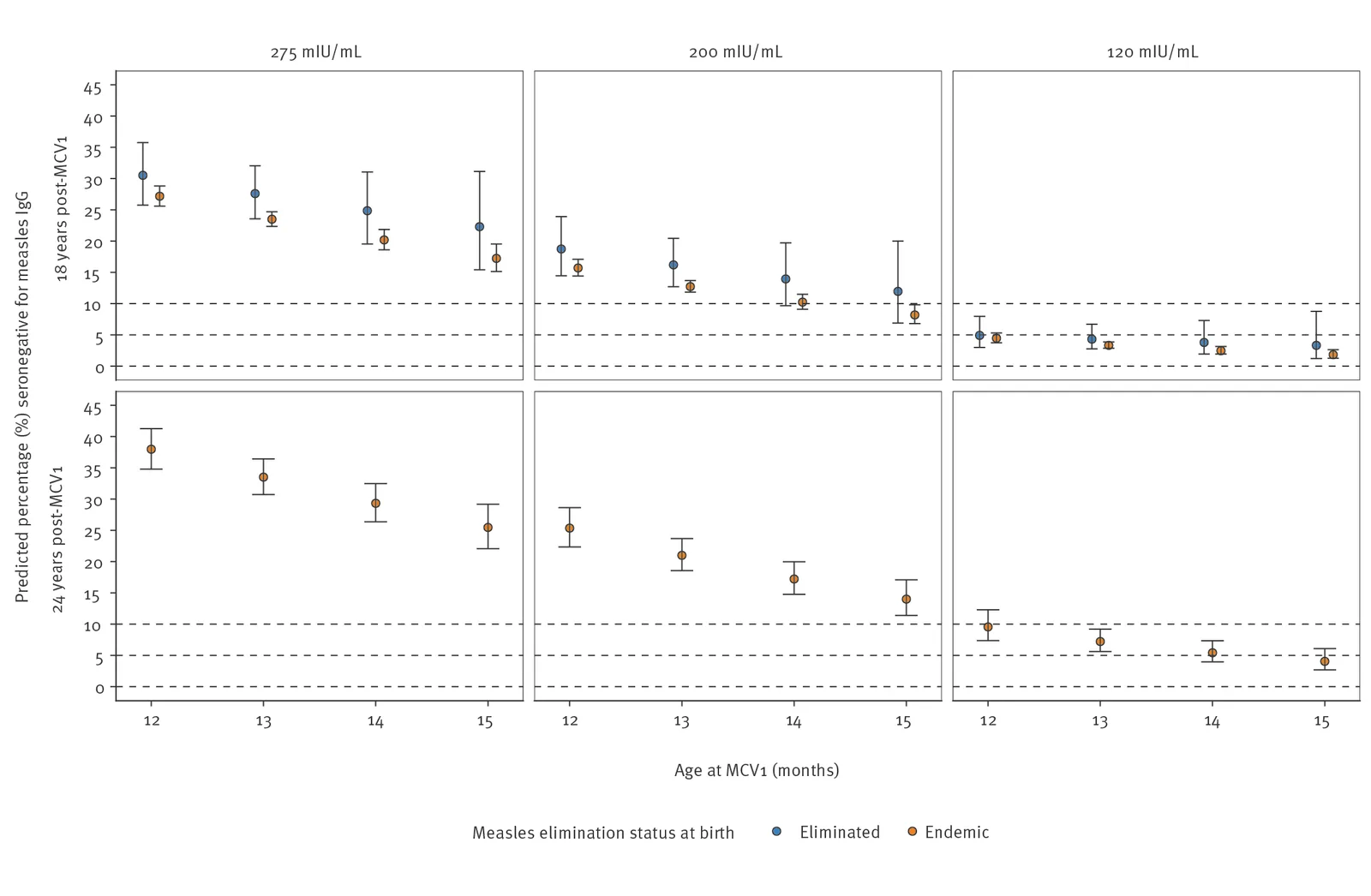
Predicted percentage seronegative for measles IgG for twice-vaccinated females at 18 and 24 years after first dose of measles-containing vaccine, by age at first dose, measles elimination status at birth and seronegativity thresholds, Ontario, Canada, 2014–2016 (n = 10,766)

IgG: immunoglobulin G; mIU/mL: milli-international units per millilitre; MCV1: first dose of measles-containing vaccine.

Predicted percentage seronegative that are above the horizontal lines at 5–10% represent overall seropositivity below 90–95% (the often-cited herd immunity threshold for measles).

At 18 years post-MCV1, individuals born before elimination had a lower predicted percentage seronegative at each equivalent age of MCV1 than those born after elimination when using the < 275 mIU/mL and < 200 mIU/mL thresholds; however, when using the ≤ 120 mIU/mL threshold, these differences were minimal. Among individuals born before elimination at 24 years post-MCV1 (the longest follow-up period), seronegativity was higher compared with those tested at 18 years post-MCV1. For vaccination at ages 12 and 15 months, respectively, the predicted percentage seronegative ranged from 38.0% (95% CI: 34.8–41.3%) to 25.5% (95% CI: 22.1–29.2) using the < 275 mIU/mL threshold, and from 9.6% (95% CI: 7.4–12.3%) to 4.1% (95% CI: 2.7–6.1%) using the ≤ 120 mIU/mL threshold, at 24 years post-MCV1.

Using the ≤ 120 mIU/mL threshold, the predicted percentage seronegative among a population with sex parity and vaccinated with MCV1 at 12 months ranged from 2.2% (95% CI: 0.2–19.8) at 1 year post-MCV1 (approximately aged 2 years) to 4.4% (95% CI: 3.0–6.4) at 16 years post-MCV1 (approximately aged 17 years). However, for a population vaccinated with MCV1 at 15 months, the predicted percentage seronegative was comparatively lower, ranging from 1.5% (95% CI: 0.1–15.5) to 3.0% (95% CI: 1.2–7.4), at the same time points, respectively. Detailed data for predicted percentage seronegative for females by time since MCV1 are appended in Supplementary Figure S7.

### Sensitivity analyses

The sensitivity analyses yielded similar results and are appended in Supplementary Figures S8, S9, S10, S11 and Supplementary Table S5.

## Discussion

In this large study, we found that a 1-month increase in age at MCV1 (between 12 and 15 months) was associated with a 9% to 14% higher predicted mean measles IgG concentration among cohorts born before and after measles elimination, respectively. In addition, a 1-month increase in age at MCV1 (between 12 and 15 months) was associated with an OR for seronegativity ranging from 0.74 (among cohorts born before elimination and using a ≤ 120 mIU/mL threshold) to 0.87 (among cohorts born after elimination and using a ≤ 120 mIU/mL threshold). These were estimated for a cohort of two-dose recipients, controlling for sex and time since MCV1. After nearly two decades of follow-up and using the lowest seronegativity threshold (≤ 120 mIU/mL), we estimated that 5–10% of those vaccinated with MCV1 at 12 months would be seronegative, whereas delaying MCV1 vaccination to 15 months would reduce the percentage seronegative to 2–4%, depending on the measles elimination status at birth and time since MCV1 (18 or 24 years).

Our results suggest that delaying MCV1 to 15 months could reduce the percentage seronegative by 2–6% in vaccinated cohorts, offsetting in part the impact of waning immunity and supporting regions with suboptimal coverage to achieve herd immunity. These findings align with our current understanding, where children vaccinated with MCV1 at older ages show a stronger immune response, potentially due to reduced maternal antibody interference and a more mature immune system, and lend support to antibody levels being an important correlate of protection [4]. Maternal antibody interference is less likely in our sample, since absence of maternal antibodies in infants has been observed as early as 3 months of age in measles-eliminated settings [5] and by 6 months of age in low-incidence measles-endemic settings [24]. However, even in the absence of maternal antibodies, immunogenicity is higher at older ages (13–15 months) potentially due to the immune system’s maturity, which is essential for sufficient B-cell diversity, antigen presentation and T-cell assistance [4,25].

More importantly, our results align with studies on protection from measles vaccination. A systematic review found limited data on vaccine effectiveness (VE) in measles-eliminated settings, but in measles-endemic settings, one-dose VE increased by 1.5% for each month MCV1 was delayed [26]. Another systematic review found a risk ratio of 0.48 (95% CI: 0.36–0.63) for measles among individuals vaccinated at ≥ 15 months compared with 12–14 months; however, all studies were from measles-endemic settings [9]. The largest study from a measles-eliminated setting, during the 2011 Quebec outbreak, found that high school students with two doses had a VE of 93% and an attack rate of 6% when vaccinated with MCV1 at 12 months, compared with a VE of 98% and an attack rate of 2% for those vaccinated at ≥ 15 months [6]. These attack rates are comparable with the percentage seronegative (≤ 120 mIU/mL) we observed in our cohort born before elimination at 24 years post-MCV (approximately aged 23 years).

While routine MCV1 is recommended at 9 months in high-risk settings and at 12–15 months in low-risk settings, it varies from 6 months in South Africa (measles-endemic) to 18 months in Sweden and Iceland (measles-eliminated) [7,27]. In the United States, the recommended age for MCV1 changed from 12 to 15 months in 1976 because data indicated that children vaccinated at 15 months had lower measles risk compared with those vaccinated earlier [4]. However, the schedule was revised to a range of 12–15 months in 1998 since children of vaccinated mothers had fewer maternal antibodies, improving response to earlier vaccination [4]. Currently, although Canada recommends MCV1 at 12–15 months, all Canadian provinces and territories schedule MCV1 at 12 months [28].

Our study found that vaccinating at 13–15 months leads to more robust humoral immunity two decades after vaccination. However, setting-specific factors such as local measles transmission and importation risks should be considered before scheduling MCV1 to older ages, especially since most infants in measles-eliminated settings become susceptible to measles by 3 months of age as maternal measles antibodies wane [5]. In 2024, Canada reported 146 measles cases, 23% of which were linked to international travel [29]. In Ontario, from 28 October 2024 to 6 January 2026, 2,376 cases were reported, the largest outbreak since measles elimination was achieved in 1998 [23]. Infants and young children were disproportionately affected, with 6.1% of cases occurring in infants aged < 1 year and 18.4% in children aged 1–4 years, of whom 100.0% and 96.1% were unvaccinated, respectively [23]. While these factors may favour vaccination at 12 months, age at MCV1 may influence lifelong measles immunity, regardless of later revaccination, as evidenced in the 2011 Quebec outbreak, which found that the effect of age at MCV1 persisted regardless of MCV2 timing [6,10]. Evidence suggests that after individuals mount a primary immune response following MCV1, revaccination may temporarily increase measles antibody concentrations if concentrations were low, but they tend to return to post-MCV1 levels [30]. Although not clear, high concentrations of antibodies from MCV1 vaccination may blunt the immune response following revaccination [30]. The measles outbreak in Canada in 2024 and 2025 was primarily driven by low vaccination coverage in sub-provincial communities, with 89% of cases reported among unvaccinated individuals [23]. This highlights that efforts to increase vaccination coverage is of highest priority to prevent future measles outbreaks [20]. Nevertheless, breakthrough infections were also reported in the outbreak in 2024 and 2025 in Ontario, where 4.7% of cases had received two or more doses; among those aged 20–39 years, the proportion of cases that were breakthrough infections and vaccinated with two or more doses is highest at 15.6% [23]. This suggests that secondary vaccine failure contributed to the outbreak, albeit to a much lesser extent than low vaccination coverage.

Our study has several limitations. Although a 1-month increase in age at MCV1 (between 12 and 15 months) was associated with a lower OR for seronegativity among those born after elimination, the association was not statistically significant. This may be due to the smaller sample size of this cohort (n = 1,426) compared with those born before elimination (n = 9,340), which probably limited our statistical power. The risk of measles among seronegative vaccinated individuals is unclear because evidence for the correlate of protection is limited, therefore we conducted analyses with various seronegativity thresholds [15]. Although neutralising antibody titres could not be directly measured because of the retrospective nature of our study, previous research has shown a moderately strong correlation (Spearman ρ = 0.71–0.79) between Euroimmun measles IgG ELISA test results and neutralising antibody titres measured by pseudotype-based virus neutralisation assay [31]. In addition, without an immunisation registry in Ontario, we heavily relied on general immunisation billing codes to determine vaccination status, which have 83% sensitivity and 81% specificity [17]. However, our algorithm classified MCV1 vaccinations concurrent with the recommended schedule. Additionally, measles infection history was not available for each individual during the study period, since not every individual was tested for measles IgM and RT-PCR. Another limitation is that our study only measured antibody concentrations, and it is not clear if those who are seronegative might be protected through cell-mediated immunity or IgA antibodies in the mucosa. Finally, the longest follow-up in our study and others was until adolescence and early adulthood, and further waning immunity may increase susceptibility and the risk of future outbreaks [10].

## Conclusion

Our study suggests that delaying MCV1 to 15 months of age may decrease the percentage of seronegative individuals by up to 6%, potentially increasing the likelihood of maintaining herd immunity. This study adds to the limited evidence available in measles-eliminated settings. Further research with longer follow-up is needed to examine the effect of MCV1 on measles immunity and VE to assess if the current schedule of providing MCV1 at 12 months is sufficient to protect vaccinated individuals over their lifetime and ensure herd immunity in measles-eliminated settings.

## Data Availability

The dataset from this study is held securely in coded form at ICES. While legal data sharing agreements between ICES and data providers (e.g., healthcare organisations and government) prohibit ICES from making the dataset publicly available, access may be granted to those who meet pre-specified criteria for confidential access, available at www.ices.on.ca/DAS (email: das@ices.on.ca). The full dataset creation plan and underlying analytic code are available from the authors upon request, understanding that the computer programmes may rely upon coding templates or macros that are unique to ICES and are therefore either inaccessible or may require modification.

## Supplementary Data

1470000 Supplementary Material
