# Supplementary material for: The effect of age at first dose of a measles-containing vaccine on measles humoral immunity: a retrospective cohort study, Canada, 2014 to 2016
Source: Euro Surveill. 2026 Nov 12;31(45):2600105. doi: 10.2807/1560-7917.ES.2026.31.45.2600105 (PMC13768771; doi:10.2807/1560-7917.ES.2026.31.45.2600105)

This supplementary material is hosted by *Eurosurveillance* as supporting information alongside the article ‘The effect of age at first dose of a measles-containing vaccine on measles humoral immunity: a retrospective cohort study, Canada, 2014 to 2016’, on behalf of the authors, who remain responsible for the accuracy and appropriateness of the content. The same standards for ethics, copyright, attributions and permissions as for the article apply. Supplements are not edited by *Eurosurveillance* and the journal is not responsible for the maintenance of any links or email addresses provided therein.

Table S1. Description of ICES databases used to ascertain sociodemographic and health information

Database	Description
Registered Persons Database (RPDB)	Basic demographic information on persons registered under the Ontario Health Insurance Plan
Ontario Health Insurance Plan (OHIP)	Physician billing claims data
Discharge Abstract Database (DAD)	Administrative, clinical and demographic data on hospital discharges
National Ambulatory Care Reporting System (NACRS)	Hospital-based and community-based ambulatory care data, including day surgeries, outpatient and community-based clinics, and emergency department visits
Immigration, Refugees, and Citizenship Canada (IRCC) Permanent Residents	Data on immigrants who initially landed in Ontario from 1985 to 2023
Ontario Cancer Registry (OCR)	Registered persons with cancer
Canadian Organ Replacement Registry (CORR)	Organ transplant data
Ontario Drug Benefit (ODB)	Prescription drug claims
Ontario HIV dataset (HIV)	Previous diagnosis of HIV
Activity Level Reporting (ALR)	Patient activity related to cancer
New Drug Funding Program (NDFP)	Data on publicly funded cancer drugs

Table S2. Algorithm to identify MCV1 between ages 12-15 months for main and sensitivity analysis (algorithm with lower specificity and higher sensitivity) among individuals born in 1992-2015

**MCV1 vaccination period**	**Recommended age (months) for MCV1**	**Concurrent vaccines by recommended age**		**Type of billing code**	**Age range searched (months)**	**Algorithm to classify age (months) at MCV1 vaccination**	
		12 months of age	15 months of age			Main analysis	Sensitivity analysis
**Sept. 2011-2016**	12	MenC-C[*] PneuC 13	Varicella	Specific[§]	12.0-14.9	First specific billing code closest to 12 months of age	First specific billing code closest to 12 months of age
**August 2011-2016**	12	MenC-C[*] PneuC 13[‡]	Varicella	General[ǀ]	12.0-14.9	≥3 general billing codes on the same age (months) and no other general codes within age range	First general code closest to 12 months of age
**2005-July 2011**	12	MenC-C[*]	PneuC 7[†] Varicella	General[ǀ]	12.0-14.9	≥2 billing general codes on the same age (months) and no other general codes within age range	First general billing code closest to 12 months of age
**1993-2004**	12	None	None	General[ǀ]	12.0-14.9	Only one general billing code<br><br>OR<br><br>Multiple general billing codes on the same age (months) and no other general billing codes within age range	First general billing code closest to 12 months of age

[*]MenC-C: Meningococcal Conjugate C
[†]PneuC 7: Pneumococcal Conjugate 7
[‡] PneuC 13: Pneumococcal Conjugate 13
[§]Specific: Immunization billing code for measles-mumps-rubella vaccine
[ǀ]General: Immunization billing code for an unspecified vaccine

Table S3. Algorithm to classify MCV2 recipients between ages 18 months and <7 years for sensitivity analysis 2

**MCV2 vaccination period**	**Recommended age for MCV2**	**Concurrent vaccines by recommended age**		**Type of billing code**	**Age range searched**	**Algorithm to classify individual as MCV2 recipients**
		**18 months of age**	**4-6 years of age**			
August 2011 - 2016	4-6 years	N/A	DTaP-IPV[‡]	General[ǀ]	4-6 years	≥2 general billing codes within age range during specified period
2005-July 2011	18 months	HiB[†]<br>DTaP-IPV[‡]	N/A	General[ǀ]	18-22 months	≥3 general billing codes within age range during specified period
1996-2004	4-6 years	N/A	DPT Polio[*]	General[ǀ]	4-6 years	≥2 general billing codes within age range during specified period

[*]DPT Polio: Diphtheria, Pertussis, Tetanus, Polio
[†]HiB: *Haemophilus influenzae* type b vaccine (Hib)
[‡] DTaP-IPV: Diphtheria, Tetanus and Acellular Pertussis vaccine (DTaP)/Inactivated Poliovirus vaccine (IPV)
[ǀ]General: Immunization billing code for an unspecified vaccine

Table S4. Proportion of serum samples as having received MCV1 and MCV2 between algorithms for main and sensitivity analysis 1 (SA1: algorithm with lower specificity and higher sensitivity) by periods defined by unique immunization schedules

**Periods defined by unique immunization schedules**	**Percentage of tested individuals with MCV1 and MCV2 classified (%)**	
	Main analysis	Sensitivity analysis
Aug. 2011 - 2016	<1	<1
2005 - July 2011	<2	<6
1993 - 2004	19.0	20.3

*Some values were presented as '<' to suppress small cell counts (n<6)

Table S5. Predicted mean measles IgG concentration (mIU/mL) and predicted percentage seronegative for measles IgG by age at MCV1 for a male vaccinated at 18 years and 24 years post-MCV1 by measles elimination status at birth and by varying thresholds for seronegativity among twice-vaccinated individuals – main analysis

Time since MCV1 (years)	Predicted mean measles IgG concentration (mIU/mL)	Predicted percentage seronegative (<275mIU/mL)	Predicted percentage seronegative (<200mIU/mL)	Predicted percentage seronegative (≤120mIU/mL)
**Born after elimination – 18 years post-MCV1**				
**12**	561 (503-625)	31.3 (24.9-38.4)	16.2 (11.2-22.9)	4.7 (2.4-9.0)
**13**	637 (576-706)	28.3 (22.7-34.8)	14.0 (9.7-19.7)	4.1 (2.2-7.7)
**14**	725 (639-822)	25.5 (19.1-33.3)	12.0 (7.5-18.5)	3.6 (1.6-8.0)
**15**	824 (696-975)	22.9 (15.3-32.9)	10.2 (5.5-18.4)	3.2 (1.0-9.2)
**Born before elimination – 18 years post-MCV1**				
**12**	672 (646-700)	27.2 (25.1-29.5)	15.3 (13.6-17.2)	4.0 (3.1-5.1)
**13**	734 (708-762)	23.6 (21.8-25.5)	12.4 (11.1-13.9)	3.0 (2.4-3.7)
**14**	802 (766-839)	20.2 (18.2-22.4)	10.0 (8.6-11.6)	2.2 (1.6-3.0)
**15**	876 (823-931)	17.3 (14.9-20.0)	8.0 (6.5-9.8)	1.6 (1.1-2.5)
**Born before elimination – 24 years post-MCV1**				
**12**	628 (593-665)	38.1 (34.5-41.7)	24.8 (21.5-28.5)	8.6 (6.4-11.5)
**13**	685 (649-724)	33.6 (30.4-37.0)	20.5 (17.7-23.6)	6.5 (4.8-8.7)
**14**	749 (704-795)	29.4 (26.1-33.0)	16.8 (14.1-19.9)	4.9 (3.4-6.9)
**15**	817 (760-879)	25.5 (21.8-29.6)	13.6 (10.9-17.0)	3.6 (2.3-5.7)

Table S6. Predicted mean measles IgG concentration (mIU/mL) and predicted percentage seronegative for measles IgG by age at MCV1 for a female vaccinated at 18 years and 24 years post-MCV1 by measles elimination status at birth and by varying thresholds for seronegativity among twice-vaccinated individuals – main analysis

Time since MCV1 (years)	Predicted mean measles IgG concentration (mIU/mL)	Predicted percentage seronegative (<275mIU/mL)	Predicted percentage seronegative (<200mIU/mL)	Predicted percentage seronegative (≤120mIU/mL)
**Born after elimination – 18 years post-MCV1**				
**12**	628 (579-682)	30.5 (25.8-35.8)	18.7 (14.5-23.9)	4.9 (3.0-8.0)
**13**	714 (665-767)	27.6 (23.6-32.0)	16.2 (12.7-20.4)	4.3 (2.8-6.7)
**14**	812 (732-900)	24.9 (19.6-31.1)	14.0 (9.7-19.7)	3.8 (1.9-7.3)
**15**	923 (793-1075)	22.3 (15.4-31.1)	12.0 (6.9-20.0)	3.3 (1.2-8.7)
**Born before elimination – 18 years post-MCV1**				
**12**	698 (678-719)	27.1 (25.6-28.8)	15.7 (14.4-17.1)	4.5 (3.8-5.3)
**13**	763 (745-780)	23.5 (22.3-24.7)	12.7 (11.8-13.7)	3.3 (2.9-3.9)
**14**	833 (805-862)	20.2 (18.6-21.9)	10.2 (9.1-11.5)	2.5 (2.0-3.1)
**15**	909 (862-959)	17.2 (15.1-19.6)	8.2 (6.8-9.8)	1.8 (1.3-2.7)
**Born before elimination – 24 years post-MCV1**				
**12**	652 (619-686)	38.0 (34.8-41.3)	25.4 (22.3-28.6)	9.6 (7.4-12.3)
**13**	712 (679-747)	33.5 (30.7-36.4)	21.0 (18.6-23.7)	7.2 (5.6-9.2)
**14**	777 (737-820)	29.3 (26.4-32.5)	17.2 (14.8-20.0)	5.4 (4.0-7.4)
**15**	849 (794-908)	25.5 (22.1-29.2)	14.0 (11.4-17.1)	4.1 (2.7-6.1)

Table S7. Observed geometric mean measles IgG concentration (mIU/mL) by age at MCV1 and time since MCV1 by measles elimination status at birth among twice-vaccinated individuals

Age at MCV1 (months)	Time since MCV1 (years)	n	Geometric mean measles IgG concentration (mIU/mL)	95% CI
**Born before elimination**				
**12**	15	29	439	316-609
**12**	16	212	453	410-500
**12**	17	927	490	464-517
**12**	18	1194	517	493-542
**12**	19	1073	498	473-525
**12**	20	870	481	453-511
**12**	21	963	451	427-477
**12**	22	622	429	401-458
**12**	23	209	397	350-450
**12**	24	15	582	346-982
**13**	15	9	828	277-2477
**13**	16	64	660	549-794
**13**	17	303	537	490-588
**13**	18	417	491	455-530
**13**	19	353	536	490-586
**13**	20	310	553	501-610
**13**	21	341	545	498-596
**13**	22	223	492	438-551
**13**	23	78	445	351-563
**13**	24	1-5	381	71-2063
**14**	15	6	414	227-756
**14**	16	35	730	571-934
**14**	17	169	593	519-677
**14**	18	210	631	562-708
**14**	19	191	563	500-633
**14**	20	169	639	556-734
**14**	21	187	524	463-592
**14**	22	112	509	432-600
**14**	23	43	527	394-704
**14**	24	1-5	NA	NA
**Born after elimination**				
**12**	4	1-5	NA	NA
**12**	5	6	504	130-1951
**12**	6	8	787	468-1323
**12**	7	1-5	NA	NA
**12**	8	1-5	NA	NA
**12**	9	1-5	NA	NA
**12**	10	1-5	NA	NA

**12**	11	1-5	NA	NA
**12**	12	1-5	NA	NA
**12**	13	16	569	414-784
**12**	14	74	470	395-559
**12**	15	255	514	465-568
**12**	16	245	532	479-591
**12**	17	337	445	405-488
**12**	18	38	514	393-672
**13**	5	1-5	NA	NA
**13**	6	1-5	NA	NA
**13**	8	1-5	NA	NA
**13**	10	1-5	NA	NA
**13**	11	1-5	NA	NA
**13**	13	7	554	237-1295
**13**	14	26	778	542-1117
**13**	15	70	600	494-729
**13**	16	80	559	457-683
**13**	17	94	614	516-732
**13**	18	13	444	284-695
**14**	6	1-5	NA	NA
**14**	7	1-5	NA	NA
**14**	8	1-5	NA	NA
**14**	13	1-5	203	82-503
**14**	14	12	574	365-903
**14**	15	38	589	441-786
**14**	16	27	675	479-951
**14**	17	39	514	380-695
**14**	18	1-5	395	33-4778

Some values are presented as NA due to suppressed small cell counts (n=1-5).

Figure S1. Distribution of measles IgG concentration by age at MCV1 and measles elimination status at birth

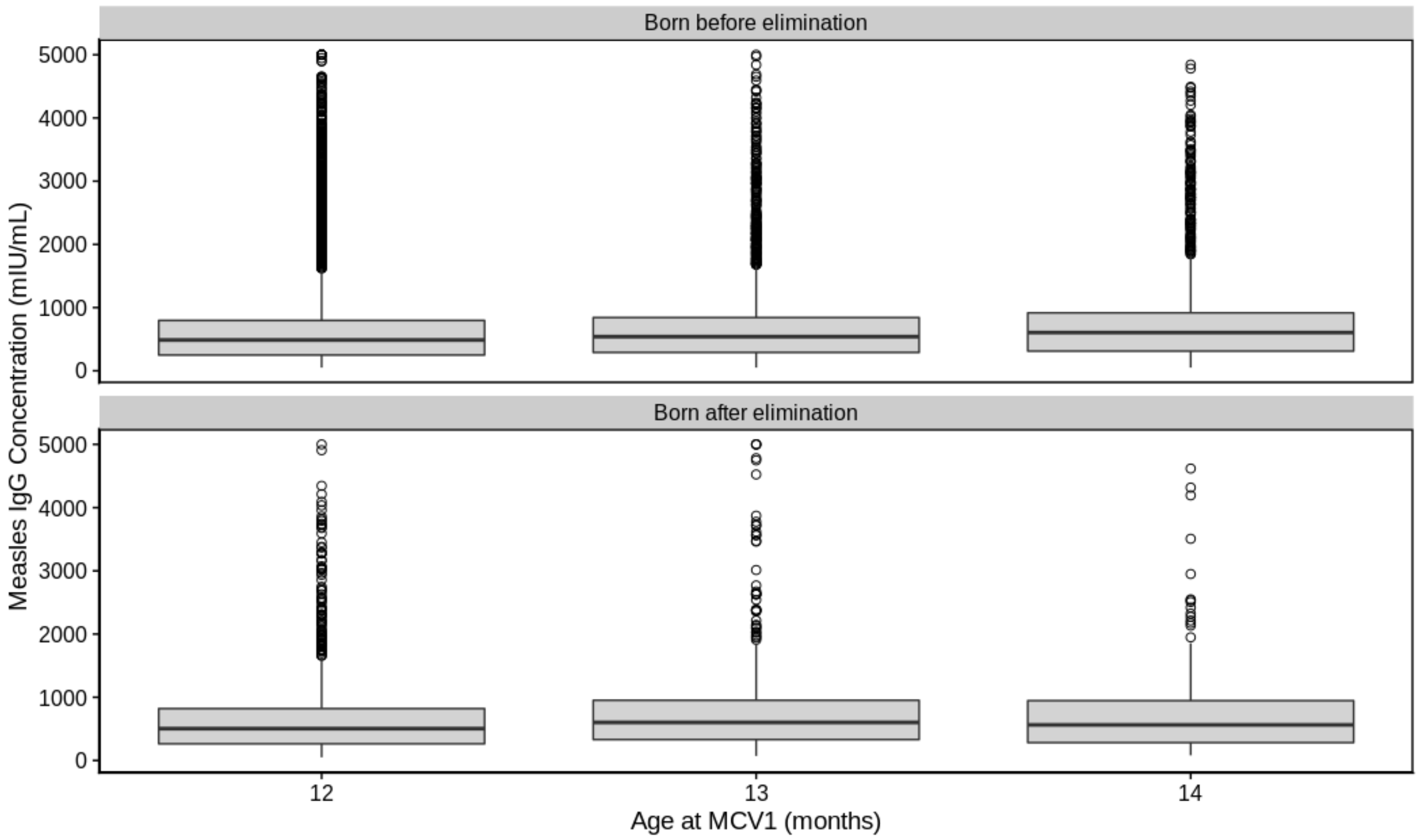

Figure S2. Excluded measles IgG test results – main analysis

377,473 Measles IgG Test Results

Excluded **366,707** Measles IgG Test Results

**Number of samples excluded**	**Description**
85	Tested positive or indeterminate for measles PCR during study period
279	Tested reactive, low reactive, or indeterminate for measles IgM during study period
235	Aged <12 months
34	One sample within a duplicate pair with identical Ontario health card number, test date, and quantitative measles IgG test result
1,522	Both samples within a duplicate pair with identical Ontario health card number and test date, but different quantitative measles IgG test result
25,843	Subsequent (e.g., second, third) measles IgG test results among individuals with multiple measles IgG test results
24	Samples that were retested and had two different quantitative measles IgG test results
311,140	MCV1 not identified
4,025	Born before 1992
129	Born outside of Canada
592	Immunocompromised
8	Time since MCV1 < 0
22,791	Not identified as MCV2 recipient based on general immunization billing codes

10,766 Measles IgG Test Results

Figure S3. Excluded measles IgG test results for sensitivity analysis 1 (SA1): Algorithm with lower specificity and higher sensitivity

377,473 Measles IgG Test Results

Excluded **365,841** Measles IgG Test Results

Number of samples excluded	Description
85	Tested positive or indeterminate for measles PCR during study period
279	Tested reactive, low reactive, or indeterminate for measles IgM during study period
235	Aged <12 months
34	One sample within a duplicate pair with identical Ontario health card number, test date, and quantitative measles IgG test result
1,522	Both samples within a duplicate pair with identical Ontario health card number and test date, but different quantitative measles IgG test result
25,843	Subsequent (e.g., second, third) measles IgG test results among individuals with multiple measles IgG test results
24	Samples that were retested and had two different quantitative measles IgG test results
306,682	MCV1 not classified
4,576	Born before 1992
169	Born outside of Canada
786	Immunocompromised
9	Time since MCV1 < 0
25,597	Not classified as MCV2 recipient

11,632 Measles IgG Test Results

Figure S4. Excluded measles IgG test results for sensitivity analysis 2 (SA2): Cohort including individuals not classified as MCV2 recipients using billing codes

377,473 Measles IgG Test Results

Excluded **343,916** Measles IgG Test Results

**Number of samples excluded**	**Description**
85	Tested positive or indeterminate for measles PCR during study period
279	Tested reactive, low reactive, or indeterminate for measles IgM during study period
235	Aged <12 months
34	One sample within a duplicate pair with identical Ontario health card number, test date, and quantitative measles IgG test result
1,522	Both samples within a duplicate pair with identical Ontario health card number and test date, but different quantitative measles IgG test result
25,843	Subsequent (e.g., second, third) measles IgG test results among individuals with multiple measles IgG test results
24	Samples that were retested and had two different quantitative measles IgG test results
311,140	MCV1 not identified
4,025	Born before 1992
129	Born outside of Canada
592	Immunocompromised
8	Time since MCV1 ≤ 0

33,557 Measles IgG Test Results

Figure S5. Age and sex distribution of sample by measles elimination status at birth

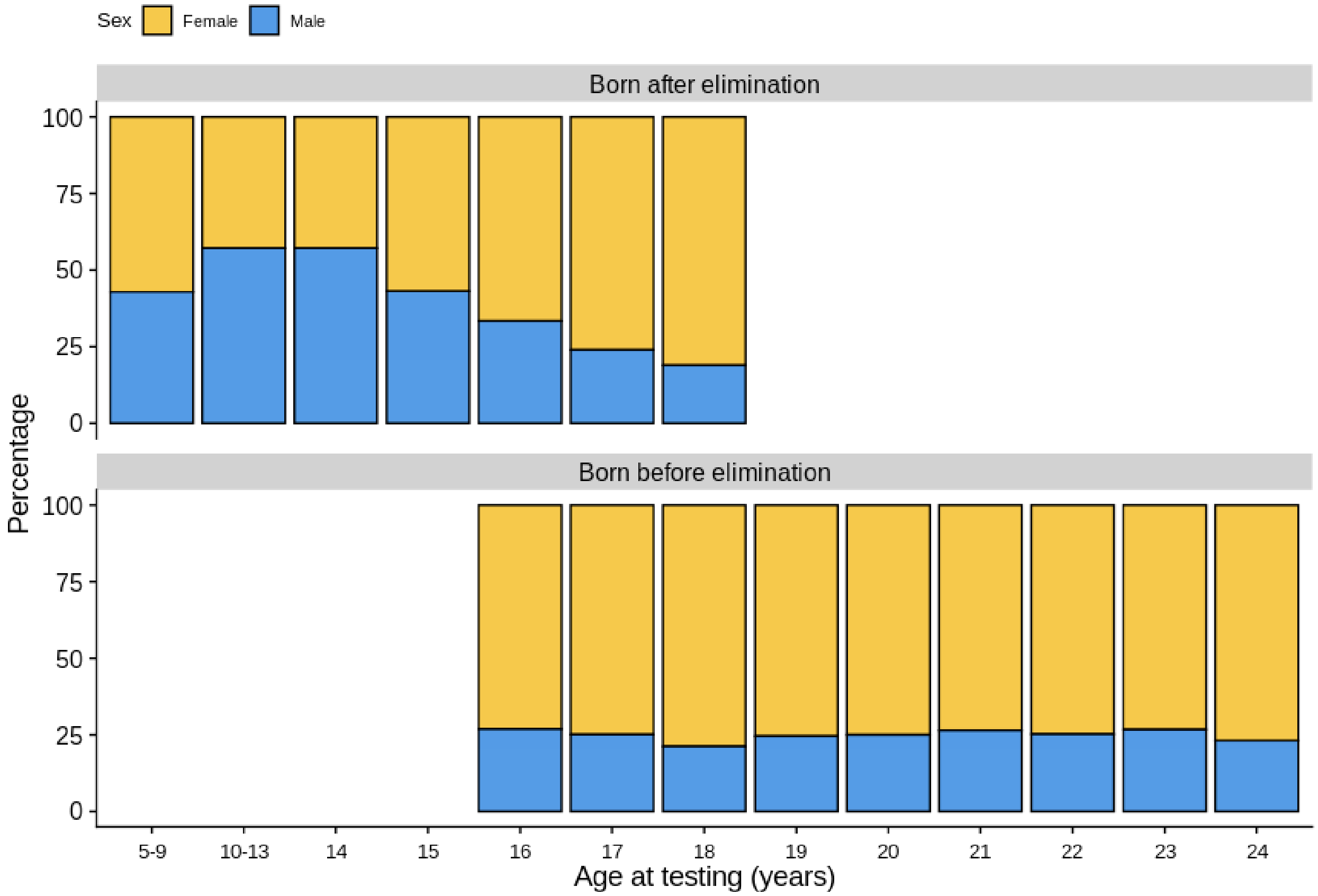

*Ages were combined to suppress small cell counts (n<6)

Figure S6. Age at MCV1 by neighborhood-level income quintile

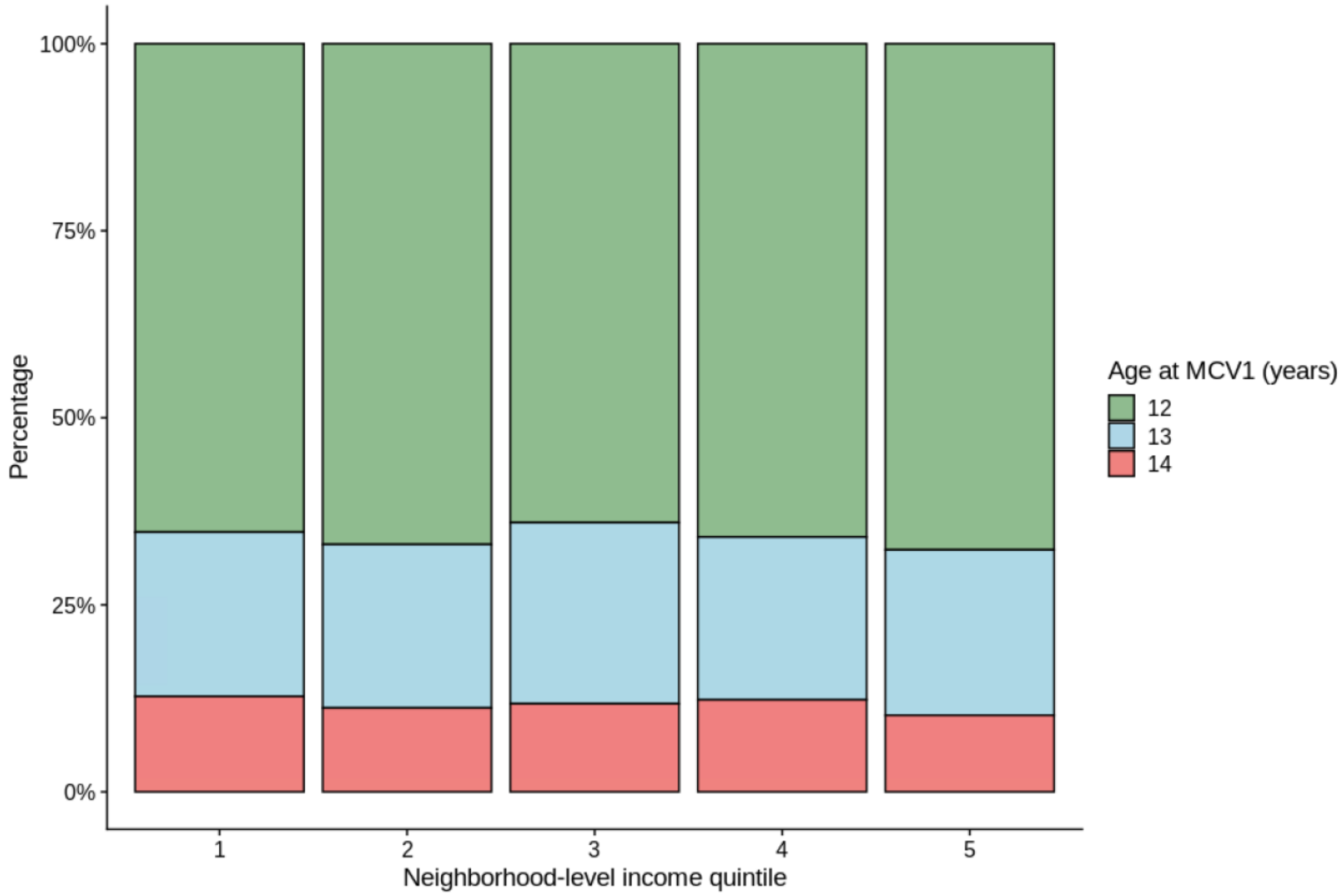

*Note: Neighborhood-level income increases from lowest income (1) to highest income (5) quintile.

Figure S7. Predicted percentage seronegative for measles IgG by age group for population with sex parity born after elimination by age at MCV1, thresholds for seronegativity, and analysis type among twice-vaccinated individuals

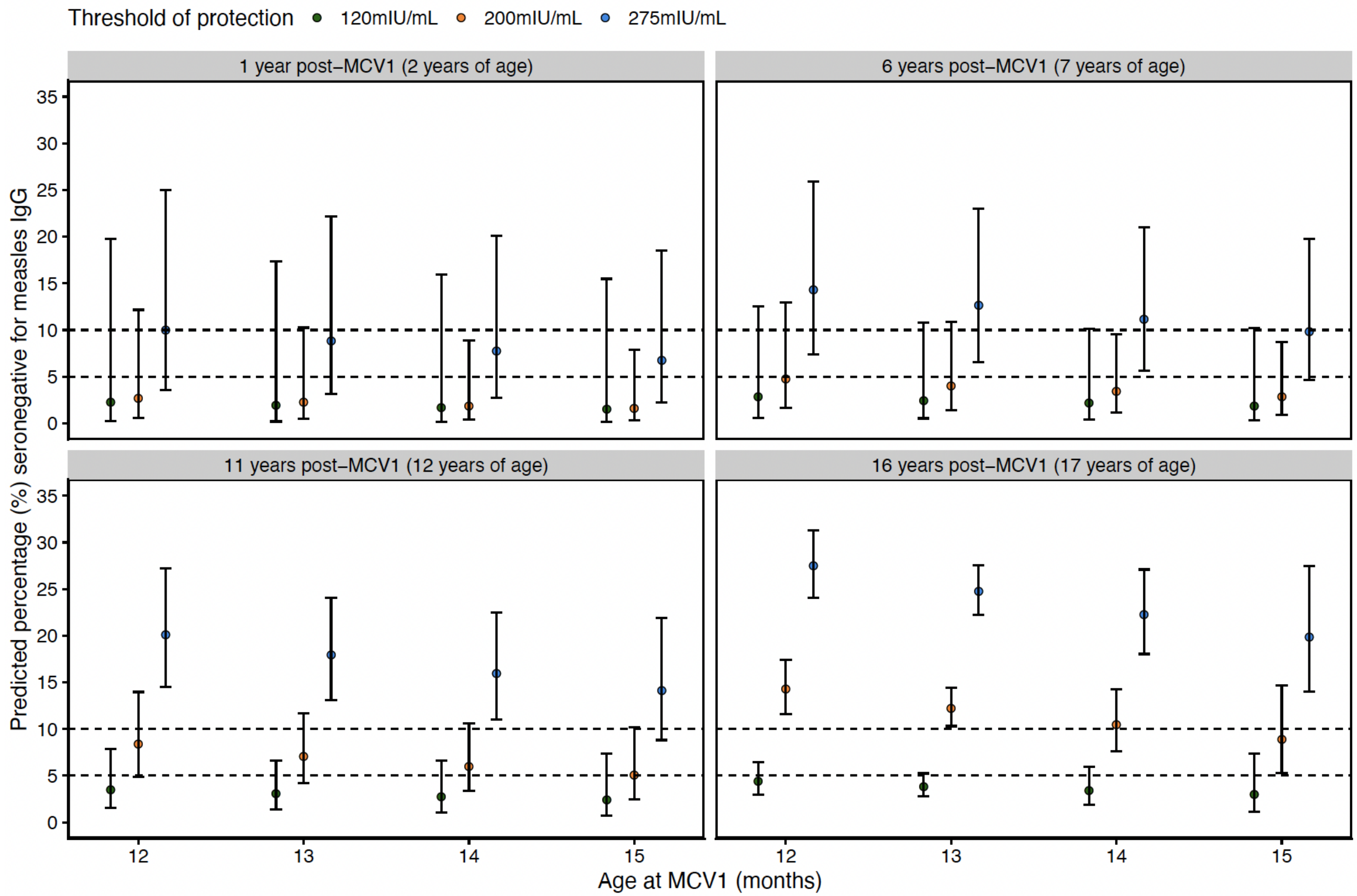

Figure S8. Predicted mean measles IgG concentration (mIU/mL) for females at 18 and 24 years post-MCV1, by age at MCV1, measles elimination status at birth, and analysis type

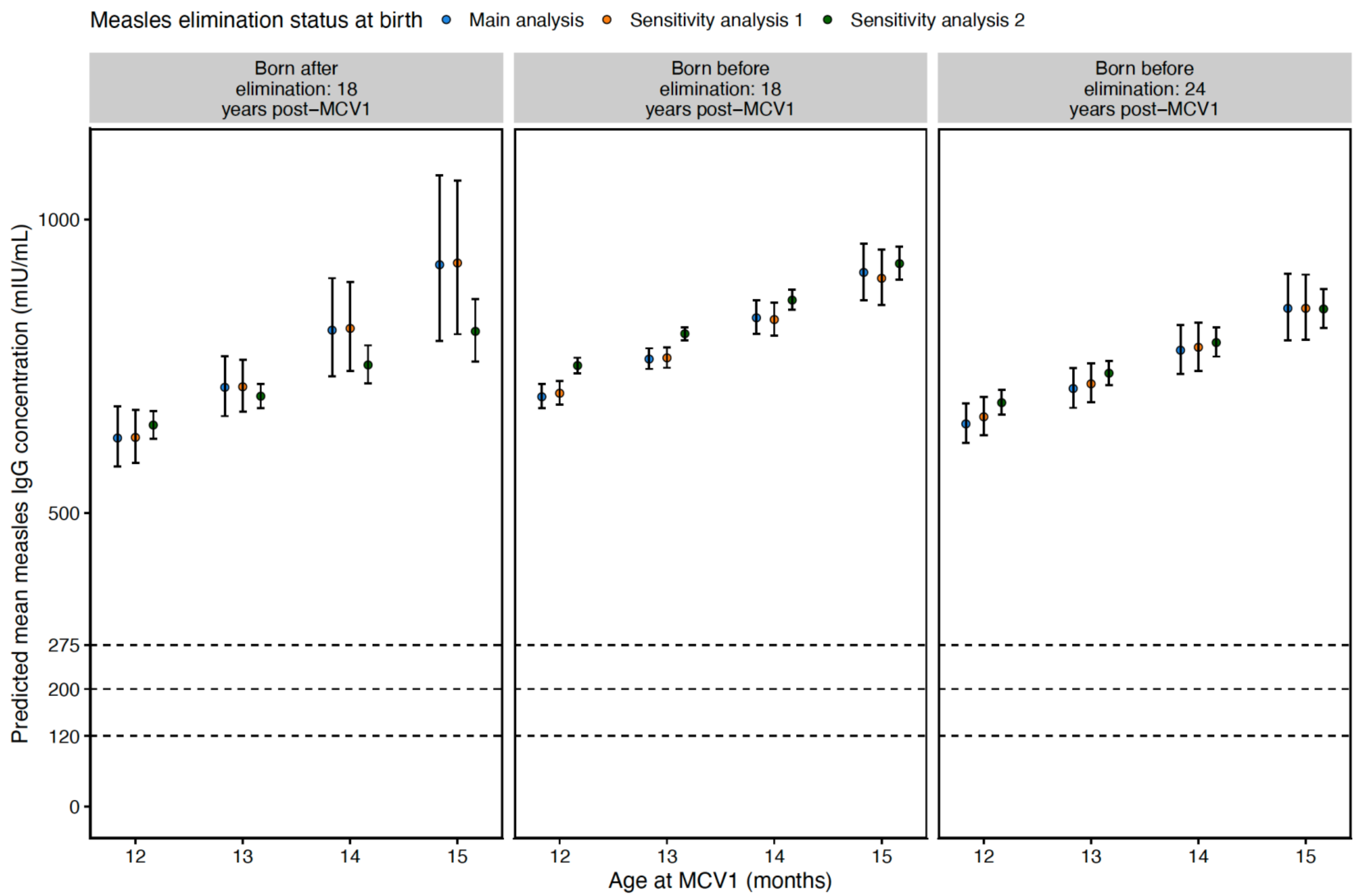

Figure S9. Predicted percentage seronegative for measles IgG for females at 18 and 24 years post-MCV1, by age at MCV1, measles elimination status at birth, thresholds of protection, and analysis type

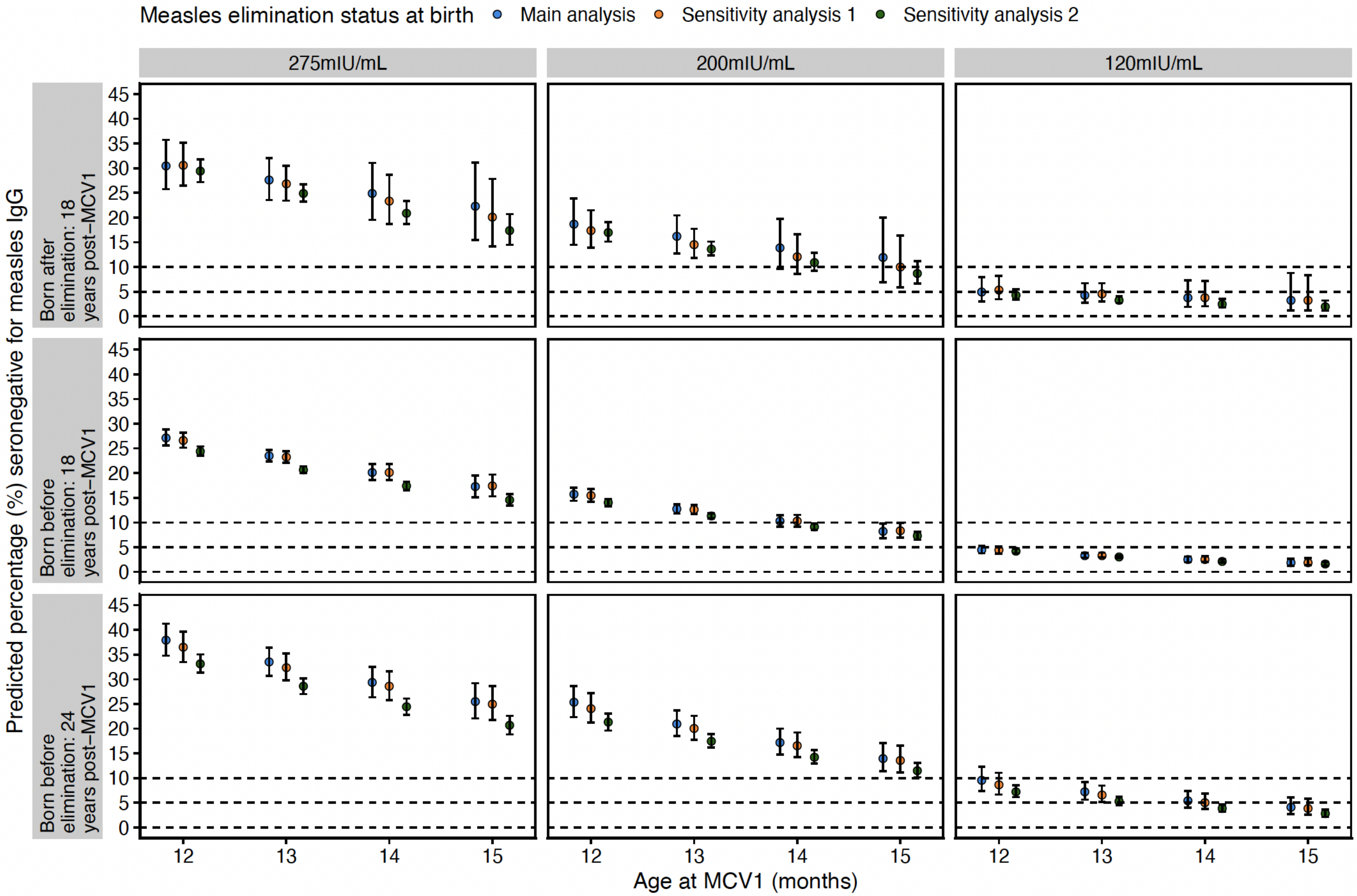

Figure S10. Predicted measles IgG concentration (mIU/mL) and 95% confidence interval for twice-vaccinated males at 18 and 24 years post- first dose of measles-containing vaccine (MCV1) by age at MCV1 and measles elimination status at birth

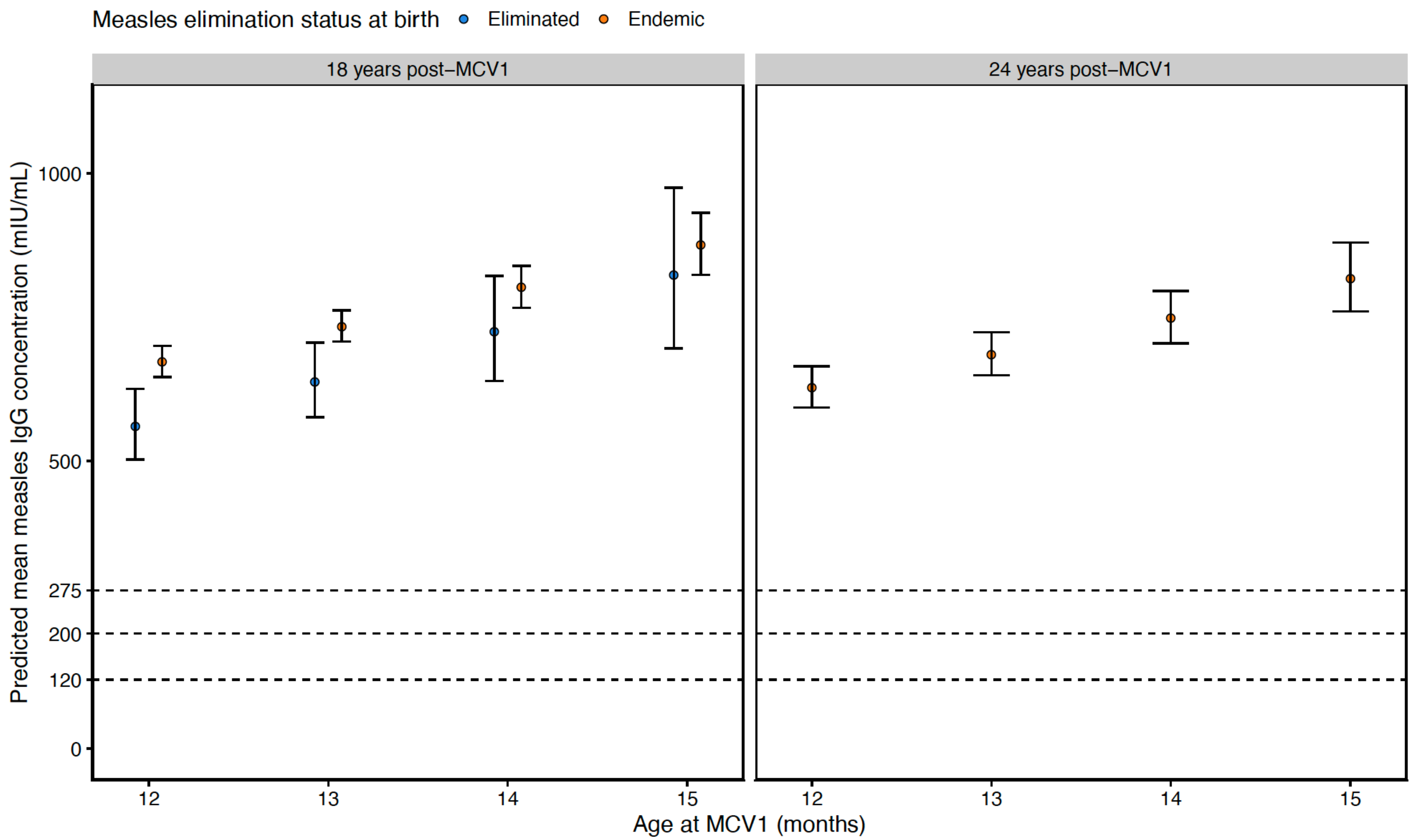

Figure S11. Predicted percentage seronegative and 95% confidence interval for measles IgG for twice-vaccinated males at 18 and 24 years post-first dose of measles-containing vaccine (MCV1) by age at MCV1, measles elimination status at birth, and seronegativity thresholds (<275mIU/mL, <200mIU/mL, ≤120mIU/mL)

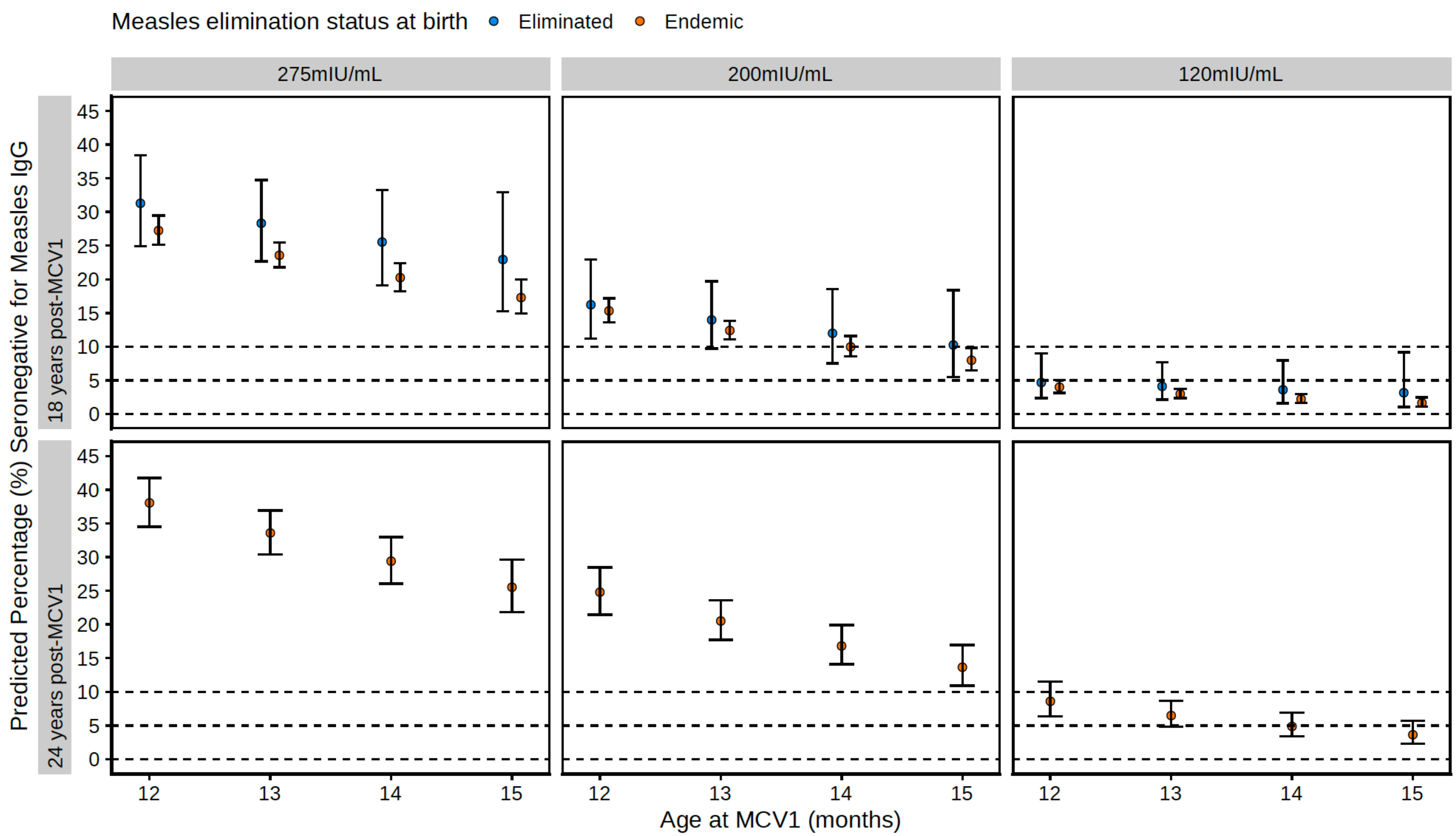

Supplement: Supplementary file 1 [file 26-00105_BOLOTIN_Supplement.pdf]
